# Paraburkholderia Xenovorans Strain LB400 Significantly Decreased Volatilization of Polychlorinated Biphenyls (PCBs) from Freshwater and Saline Sediments

**DOI:** 10.1021/acsestwater.5c00423

**Published:** 2025-09-19

**Authors:** David J. Ramotowski, Andres Martinez, Rachel F. Marek, Keri C. Hornbuckle, Timothy E. Mattes

**Affiliations:** † Department of Civil and Environmental Engineering, 4083 University of Iowa, 4105 Seamans Center, Iowa City, Iowa 52242, United States; ‡ IIHR-Hydroscience and Engineering, 100 C. Maxwell Stanley Hydraulics Laboratory, University of Iowa, Iowa City, Iowa 52242, United States

**Keywords:** bioremediation, polychlorinated biphenyls, passive sampling, microcosms, sediment, Paraburkholderia xenovorans LB400, biphenyl dioxygenase

## Abstract

Airborne PCB emissions from contaminated sediments pose a public health risk and are frequently cited as a concern for communities located near PCB-contaminated bodies of water. We assessed the potential to decrease the emissions of lower-chlorinated (LC)-PCBs (<3 chlorines) through bioaugmentation with aerobic PCB-degrading *Paraburkholderia xenovorans* strain LB400 in laboratory microcosms using historically PCB-contaminated sediments from a wastewater lagoon (Altavista, VA; AVL) and an estuary (New Bedford Harbor, MA; NBH). We compared the impact of nonshaken vs shaken conditions on airborne PCBs in LB400-bioaugmented AVL sediment (51% LC-PCBs) to better replicate field conditions. After 35 days, airborne LC-PCBs decreased by 54% in nonshaken bioaugmented AVL sediments, compared to a 60% decrease in shaken bioaugmented sediments. Bioaugmenting LB400 into unshaken NBH sediments (44% LC-PCBs) significantly decreased airborne LC-PCBs by 50% over 35 days. Biphenyl dioxygenase gene (*bphA*) abundance decreased by several orders of magnitude after 16 days in all experiments, demonstrating a potential decrease in treatment effectiveness over time. These novel findings demonstrate that LB400 effectively degrades LC-PCBs with varying profiles over a range of environmentally relevant mixing scenarios. Further treatment delivery development has the potential to protect nearby communities from PCB exposure, decrease health risks, and improve quality of life.

## Graphical abstract

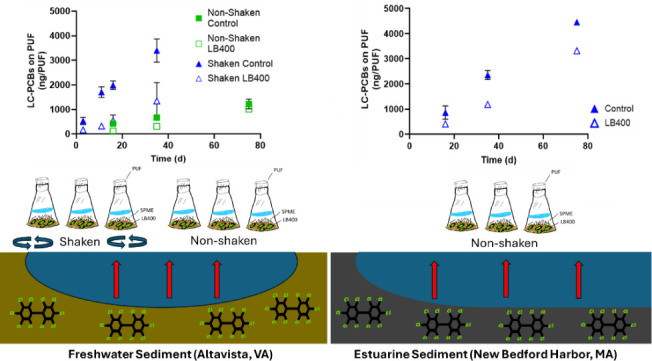

## Introduction

Polychlorinated biphenyls (PCBs) are human-made organic chemicals well-known for their toxicity and persistence in the environment.^1,3^ PCBs were marketed as Aroclors in the United States,^2,3^ and there are 209 different PCB congeners, further divided into 10 homologue groups based on the number of chlorine atoms.^4,5^ Because of their hydrophobicity, PCBs are often found sorbed to organic material present in sediments,^6–8^ but can also dissolve into water and volatilize into air. Human PCB exposure can occur through eating contaminated fish, or through inhaling airborne PCBs.^1,2,7,9^ Of particular concern for inhalation are the more volatile lower-chlorinated PCBs (LC-PCBs), defined here as congeners with three chlorine substituents or less. PCB-contaminated sediment is frequently removed by dredging and placing it into a chemical waste landfill, however, this is invasive, expensive, and may release more PCBs into the air.^10–13^

Removing PCBs with bacteria, i.e., bioremediation, is a promising *in situ* approach to managing PCB-contaminated sediments.^11,14–18^ For instance, *Paraburkholderia xenovorans* LB400,^19^ a microorganism well-known for its ability to degrade LC-PCBs,^20,21^ through aerobic oxidation with the enzyme biphenyl dioxygenase,^6,13,19,22–26^ has been used in numerous PCB bioremediation studies.^13,16,22,23,25,27–30^ Diverse PCB-degrading aerobes include strains of *Rhodococcus* ^31,32^ and *Pseudomonas*, among others.^33–35^ Microbial biodegradation of PCBs occurs through two microbial pathways: aerobic oxidation and anaerobic reductive dechlorination.^11,36–39^ Anaerobic organohalide-respiring bacteria (OHRB) transform higher-chlorinated (HC)-PCBs into LC-PCBs,^25,40,41^ many of which can be subsequently oxidized by aerobic PCB-degrading bacteria. Anaerobic PCB-dechlorinating OHRB include *Dehalococcoides* sp.^42,43^ and *Dehalobium*,^44,45^ which are more prevalent in freshwater and saline systems, respectively.^46^ For this reason, a combined *in situ* treatment using anaerobic and aerobic bacteria is a promising alternative solution, however, bioaugmentation strategies using aerobic PCB degraders need to be developed further.^13,16,23,25^

Quantitative reactive transport models have been useful for capturing and better interpreting the complexity of key physical, chemical, and biological mechanisms governing contaminant biodegradation in sediments.^47–50^ However, PCB bioremediation studies generally neglect PCB volatilization, and rarely take advantage of modeling and passive sampling approaches to detect PCBs in the aqueous^23,51^ and airborne phases.^23^ A recent study, which used polyurethane foam (PUF) and solid phase microextraction (SPME) to quantify PCBs in air and water, showed that bioaugmentation with suspended LB400 significantly decreased airborne PCBs from a contaminated sediment by 57%.^23^ Experimental conditions for aerobic LC-PCB biodegradation were optimized in that study by shaking, which can enhance PCB mass transfer from sediment to water and increase oxygen mass transfer rates into water. Determining the effects LB400 bioaugmentation on PCB volatilization in sediments under nonturbulent (i.e., nonshaken) conditions represents a more realistic approximation of sediment environments.

The impact of LB400 bioaugmentation on LC-PCB volatilization has only been tested in sediment from a quiescent decommissioned overflow lagoon in Altavista, VA (AVL). AVL was historically contaminated with Aroclor 1248, a PCB mixture composed of 23% LC-PCBs.^52^ PCB-contaminated sediments with higher LC-PCB content could be well-suited for LB400 bioaugmentation applications that aim to decrease PCB volatilization and subsequent human exposure by inhalation. For instance, New Bedford Harbor, Massachusetts (NBH) is a brackish estuary contaminated mostly with Aroclors 1242 and 1016 containing 70% and 60% LC-PCBs, respectively.^24,42,45,53,54^ NBH is known for having high sediment PCB concentrations and high airborne PCB emissions,^7^ which have elevated health risks among the nearby population.^9^

The purpose of this study was to determine how variable mixing conditions and LC-PCB content impact aqueous phase and airborne PCB levels in two different PCB-contaminated sediments bioaugmented with biphenyl-grown strain LB400. We conducted two experiments (shaken and nonshaken) with AVL sediment and one nonshaken experiment with NBH sediment. Our objectives were to determine if (1) LB400 bioaugmentation decreases LC-PCB volatilization in AVL sediment under nonshaken conditions, (2) LB400 bioaugmentation decreases aqueous phase and airborne PCBs in NBH sediment, which contains a different LC-PCB mixture than AVL, and (3) adapt and apply a refined PCB reactive transport model to assist in interpreting experimental data under nonshaken conditions. Our results represent an important step toward implementing PCB bioremediation at field scale in a safe, practical way.

## Methods and Materials

### Sediment

Sediments were obtained from Altavista, Virginia^23,24^ (AVL) and New Bedford Harbor, Massachusetts^55^ (NBH; Figure S1), homogenized using a power drill and paint mixer attachment,^23,24^ and stored at 4 °C. PCBs were extracted from NBH sediment (Section S1 **)** and measured as described below. The average total PCB concentration (ng PCBs per g dry sediment) was 6350 ± 85.4 ng g^–1^ in AVL^23^ and 59000 ± 824 ng g^–1^ in NBH. AVL sediment contained 51% LC-PCBs (3250 ng g^–1^),^56^ and NBH sediment contained 44% LC-PCBs (25800 ng g^–1^). NBH sediment containing the highest total and LC-PCB concentrations (location INT_222) (Figure S1) was used in experiments.

### Bacterial Culture, Growth Medium, and Chemicals

*Paraburkholderia xenovorans* LB400 (ATCC #43038)^57,58^ was grown in liquid K1 medium^22,59^ (recipe in SI Section S1) using biphenyl crystals (0.76–1.5 g/L; TCI America, Portland, Oregon) as the sole carbon and energy source, at 30 °C in a shaking incubator set to 150 rpm. At midexponential phase (optical density at 600 nm (OD_600_) of 0.8–1.0 measured with a Cary 50 Bio UV–visible Spectrophotometer (Varian; Palo Alto, California)), cells were harvested by centrifuging at 5000*g* for 15 min, washed once with 50–100 mL fresh K1 media, and resuspended in K1 media (OD_600_ of ∼1.0–1.2). We diluted the LB400 suspension to a final OD_600_ of 0.6 in 100 mL of K1 media in each microcosm to normalize the biomass used in all experiments.

### PCB Measurements in Air and Water

Cylinders (height 2.5 cm, radius 1.9 cm) of polyurethane foam (PUF; Tisch Environmental Inc., Cleves, Ohio) were used for measuring airborne PCBs.^6^ Solid phase microextraction fibers (SPME; ∼2.5 cm segments, Sigma-Aldrich, St. Louis, Missouri) coated with a 10 μM layer of polydimethylsiloxane were used for measuring freely dissolved aqueous PCBs through direct immersion. SPME fibers are free of organic solvents, quickly reach equilibrium, and are sensitive to small changes in PCB concentration.^60,61^

All PUF cylinders and SPME fibers were cleaned to remove background PCB contamination before use. PUF cylinders were cleaned using an Accelerated Solvent Extractor (ASE; settings described in Section S1) with a 1:1 ratio of acetone:hexane, and SPME fibers were cleaned by soaking in hexane for ∼24 h. Each PUF was wrapped in aluminum foil, and SPME fibers were wrapped together in aluminum foil and stored at −10 °C until use.

### Microcosm Setup, Experimental Design, and Sampling Procedures

Erlenmeyer flasks (250 mL), combusted overnight (450 °C) to remove background PCB contamination, were used for the microcosm body.^6^ We used a 1:10 ratio of sediment:liquid, with 10 g sediment and 100 mL of K1 medium (control), or 100 mL of K1 medium with LB400 (OD_600_ ∼ 0.6; treatment). We added ∼30 cm of SPME fibers, placed a PUF cylinder in the neck of the flask, and covered with aluminum foil to minimize air exchange.

Three experiments were designed to quantify the effects of shaking and LB400 bioaugmentation on PCB volatilization in AVL and NBH sediments. The three experiments were: adding LB400 to AVL sediment with shaking (AVL_S), adding LB400 to AVL sediment without shaking (AVL_NS), and adding LB400 to NBH sediment without shaking (NBH_NS) for 35, 75, and 75 days, respectively (Figure S2). Sampling times were days 3, 11, 16, and 35 for AVL_S, and days 16, 35, and 75 for AVL_NS and NBH_NS. Each set of microcosms was prepared in triplicate and sampled sacrificially. A total of 24 microcosms were prepared for AVL_S and 18 for AVL_NS and NBH_NS. AVL_S microcosms were shaken at 150 rpm. All microcosms were incubated at room temperature (∼21 °C).

At each sampling time, PUF were removed, wrapped in aluminum foil, and stored at −10 °C to prevent volatilization. SPME fibers were sampled by pouring the liquid/sediment from the microcosm into a 100 mL Petri dish, removing the fibers with tweezers, and washing with Optima water (Thermo Fisher Scientific, Waltham, Massachusetts). Fibers were measured and placed into a 2 mL GC vial fitted with a glass insert. Vials were filled with 1 mL hexane to desorb PCBs and stored at −20 °C.

For DNA extraction and qPCR analysis, sediment slurry samples (5 mL) were taken from AVL_S microcosms, while wet sediment samples (0.25 g) were taken from AVL_NS and NBH_NS microcosms. All sediment was stored at −10 °C until DNA extraction.

### PCB Extraction from PUF and SPME Samples

Airborne PCBs were extracted from PUF samples using an ASE 350 (Dionex, Sunnyvale, California),^6,7,23,24,62^ (details in Section S1 **)**. PCBs in SPME samples were extracted by placing the SPME fibers into a GC vial with a glass insert and adding hexane and internal standard (10 ng) prior to quantification.

### PCB Quantification Using GC-MS/MS

PUF and SPME samples were quantified through GC-MS/MS (7890A GC system, 7000 Triple Quad, 7693 autosampler; Agilent, Santa Clara, California) in multiple reaction monitoring mode (MRM), producing 171 individual or coeluting congener peaks for the 209 PCB congeners.^7,62^ The GC was equipped with a Supelco SBP-Octyl capillary column (Poly­(50% n-octyl/50% methyl siloxane), 30 m × 0.25 mm ID, 0.25 μm film thickness) with UHP helium as the carrier gas (0.8 mL/min) and UHP nitrogen as the collision gas (1.5 mL/min). Additional GC-MS/MS operating conditions are in Section S1.

### DNA Extraction and Quantitative PCR

DNA was extracted from microcosm sediment samples using a DNeasy PowerSoil Pro kit (Qiagen, Hilden, Germany). Sediment slurry samples were centrifuged at 5000*g* for 20 min, and the supernatant was removed prior to DNA extraction. DNA concentrations (ng/μL) were measured using the Qubit dsDNA high sensitivity assay kit and a Qubit 4 fluorometer (Thermo Fisher Scientific, Waltham, Massachusetts). The *bphA* abundance in each sample was measured with qPCR (20 μL reactions, consisting of 10 μL Power SYBR Green PCR Master Mix (Invitrogen, Waltham, Massachusetts), 10 ng template DNA, 1 μM forward and reverse *bphA* primers (Table S1), and 0.1 μL bovine serum albumin (20 mg/mL; New England Biolabs, Ipswich, Massachusetts). Plasmids containing the LB400 *bphA* were serially diluted in triplicate to create standard curves (Table S2). Additional QA/QC details are provided according to MIQE guidelines.^22,63^

### PCB Reactive Transport Modeling

We predicted transport and biodegradation of specific PCB congeners in the aqueous phase and headspace in shaken microcosms using our previously described PCB reactive transport model (RTM)^64^ (DOI: 10.25820/code.006163). The RTM was further refined to include fast and slow sorption/desorption between sediment particles and the freely dissolved phase, and the PCB fraction in sediment particles available for sorption/desorption processes. For the nonshaken experiments, a radial diffusion kinetic model was used to predict PCB release from sediment particles to porewater.^65–67^ Exchange between sediment porewater and the aqueous phase was estimated with a sediment-water mass transfer model based on PCB diffusion across the liquid boundary layer.^66,68^ To account for PCB sorption on LB400 cells, a bioavailability factor (B)^47,51^ was included. Each compartment, including both passive samplers, was described as a differential equation based on volumetric concentrations then transformed to mass per PUF and mass per cm (SPME) to facilitate comparisons between our measurements and model predictions. RTM equations and parameters are shown in Section S2.

### Quality Assurance and Control

Surrogate standards were used to evaluate the quality of PUF sample extractions. The average recoveries for the ^13^C-labeled PCBs ranged from 64 ± 14% to 92 ± 15%. Each surrogate was used to correct individual PCB congener masses within the same homologue group. PUF (*n* = 19) and SPME (*n* = 3) laboratory blanks were used to assess contamination and to calculate the limit of quantification $(\mathrm{LOQ})=\mathrm{Average}\left(\mathrm{mass}\;\mathrm{PCBs}\right)+\frac{1.96\times \mathrm{STDev}\left(\mathrm{mass}\;\mathrm{PCBs}\right)}{\sqrt{n}}$ for each PCB congener. Because PUF extractions occurred in two different locations, we calculated two LOQs, which were 28 ng and 68 ng per PUF (sum total PCBs). The LOQ for SPME was 0.13 ng per cm fiber. For all PUF and SPME samples, we dichotomized at the threshold of the LOQ by replacing any PCB congener with a value below the LOQ with zero.

Concentrations of PCBs in the air, aqueous, and sediment phases, method blanks for PUF and SPME, and masses of individual PCB congeners used in internal, surrogate, and calibration standards are published open access at 10.25820/data.007563.^69^

### Statistical Analysis

PCB concentration data in ng PCB per PUF or ng PCB cm^–1^ (SPME) was log_10_ transformed to normalize the distribution, and a series of paired, two-tailed *t*-tests was used at each time point to determine if any differences in aqueous-phase PCBs accumulated on SPME or airborne PCBs accumulated on PUF between the treatment and control were statistically significant. We used R^70^ and the Lattice package^71^ to conduct the statistical analyses. A *p*-value< 0.05 indicated a significant difference between treatment and control at that time point. RTM performance was assessed using the coefficient of correlation (*R* ^2^) between average measured values over time and corresponding model predictions. The complete set of R codes used for modeling and statistical analysis are published open access at 10.5281/zenodo.16368721.^72^

## Results and Discussion

### Bioaugmentation with Strain LB400 Decreased LC-PCB Volatilization from Non-Shaken Freshwater Sediment

Previous LB400 sediment bioaugmentation and PCB volatilization experiments were performed under shaken conditions.^23^ Shaking increases PCB release from sediment, enhances mass transfer from sediment to water, and improves oxygen transfer through diffusion. Here, we tested whether LB400 bioaugmentation decreased LC-PCB volatilization in a nonshaken system, where potentially lower oxygen and PCB bioavailability could impact LB400s ability to degrade PCBs, as compared to a shaken system.

Bioaugmentation with LB400 greatly decreased total and LC-PCB volatilization under both shaken and nonshaken conditions after 35 days, showing that LB400 is effective in a nonshaken environment like the quiescent AVL. The AVL_S treatments (1840 ± 1000 ng PCBs) accumulated 54% fewer total PCBs than the AVL_S controls (3980 ± 553 ng PCBs; *p* = 0.092), and the AVL_NS treatments (364 ± 33.2 ng PCBs) showed a 55% decrease, compared to AVL_NS controls (806 ± 9.76 ng PCBs; *p* = 0.002) (Figure 1A). LC-PCBs similarly decreased by 60% in AVL_S treatments (*p* = 0.068) and 54% in AVL_NS treatments (*p* = 0.001), compared to controls after 35 days (Figure 1B).

**Figure 1:**
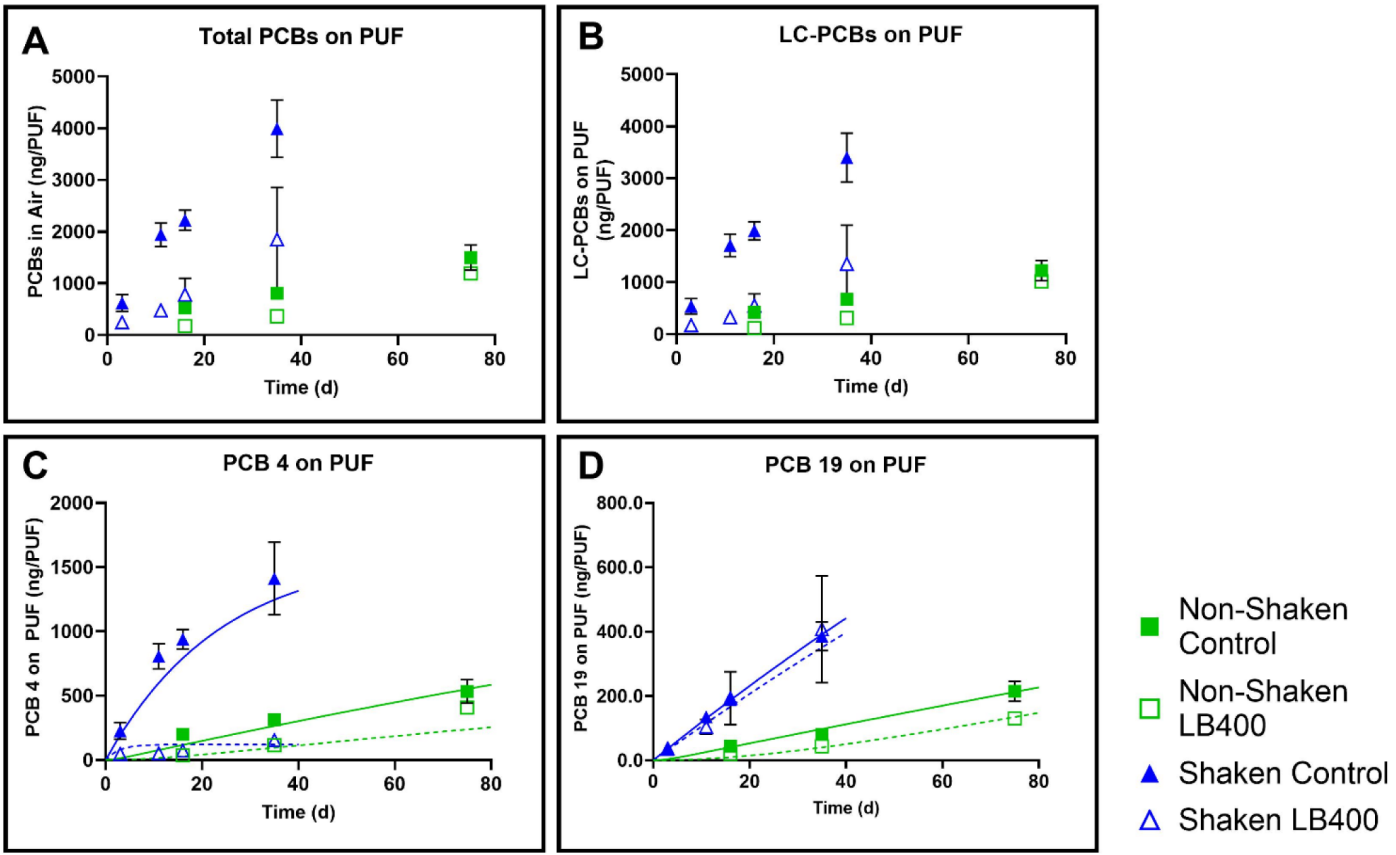
Bioaugmentation with *Paraburkholderia xenovorans*LB400 significantly decreased airborne PCBs from both shaken (AVL_S) and nonshaken (AVL_NS) Altavista sediment microcosms. Panel A: Total airborne PCBs after 35–75 days (AVL_S; blue triangles) and 75 days (AVL_NS; green squares); Panel B: LC-PCBs; Panel C: PCB 4 (2,2’-dichlorobiphenyl); Panel D: PCB 19 (2,2’,6-trichlorobiphenyl). Data points are the average of 3 replicates and the error bars represent the standard deviation. PCB reactive transport model predictions for PCB 4 and PCB 19 concentrations are shown as solid lines (control; green for nonshaken, blue for shaken) or dashed lines (LB400 bioaugmentation; green for nonshaken, blue for shaken).

We also analyzed the impact of LB400 bioaugmentation on volatilization of individual LC-PCB congeners PCB 4 and PCB 19. PCB 4 is well degraded by LB400, while double-ortho substituted PCB 19 resists biodegradation by LB400.^23^ After 35 days in bioaugmented AVL_S treatments, PCB 4 volatilization decreased by 89% (*p* = 0.0004) (Figure 1C), while PCB 19 remained almost identical to the AVL_S control (*p* = 0.986, Figure 1D). This is consistent with our previous work,^23^ which showed a highly significant decrease in PCB 4 in shaken AVL sediment treated with LB400, while the lack of decrease in PCB 19 indicates that PCB biodegradation likely did not occur. After 35 days of the AVL_NS experiment, airborne PCB 4 (Figure 1C) decreased by 63% in LB400-treated samples compared to the controls (*p* = 6 × 10^–5^). Airborne PCB 19 (Figure 1D) decreased by 46% in AVL_NS treatments (*p* = 0.019), which was unexpected since there was no significant change in AVL_S between treatments and controls. This unexpected decrease in PCB 19 in AVL_NS may have been caused by sorption of PCBs onto LB400 cells, which decreases the aqueous-phase concentration, leading to a decrease in airborne concentration.

We ran nonshaken experiments for 75 days instead of 35 days as in AVL_S because decreased oxygen mass transfer and PCB bioavailability in these nonshaken systems could slow PCB biodegradation by LB400. After 75 days, total airborne PCBs (Figure 1A) were 20% lower in the LB400-treated samples from AVL_NS (*p* = 0.137), while LC-PCBs (Figure 1B) were 17% lower (*p* = 0.191). Airborne PCB 4 (Figure 1C) only decreased by 23% after 75 days (*p* = 0.099). This suggests that LB400 activity decreased over time, which is consistent with our previous observations,^23^ and is particularly pronounced at day 75. Airborne PCB 19 was 39% lower in the AVL_NS treatment (*p* = 0.012) at day 75 compared to AVL_NS controls (Figure 1D).

Bioaugmentation with LB400 decreased aqueous phase PCBs after 35 days in the shaken treatment, but not in the nonshaken treatment, compared to controls. In AVL_S, there were 6.2 ± 1.9 ng total PCBs cm^–1^ fiber in the controls (Figure 2A) and 4.4 ± 2.6 ng cm^–1^ in the treatments after 35 days; a 29% decrease (*p* = 0.326). In AVL_NS, there were 1.4 ± 0.12 ng cm^–1^ in the controls and 2.9 ± 0.57 ng cm^–1^ in treatments after 35 days; a 94% increase (*p* = 0.015). LC-PCB trends (Figure 2B) were similar, with a 38% decrease in the AVL_S treatment, and a 213% increase in the AVL_NS treatment, compared to controls. The increased freely dissolved PCBs in treatments relative to the controls in AVL_NS was unexpected, and contradicted airborne PCB trends, which showed decreased airborne PCBs in L400 treatments. We did not observe any physical differences in the SPME fibers between the shaken and nonshaken experiments. Aqueous PCB 4 (Figure 2C) at day 35 in AVL_NS and AVL_S was significantly greater in both the controls (*p* = 0.003) and treatments (*p* = 0.004). PCB 19 (Figure 2D) was significantly different between the two controls (*p* = 8.85 × 10^–5^), but the difference between the two treatments was not significant (*p* = 0.738).

**Figure 2:**
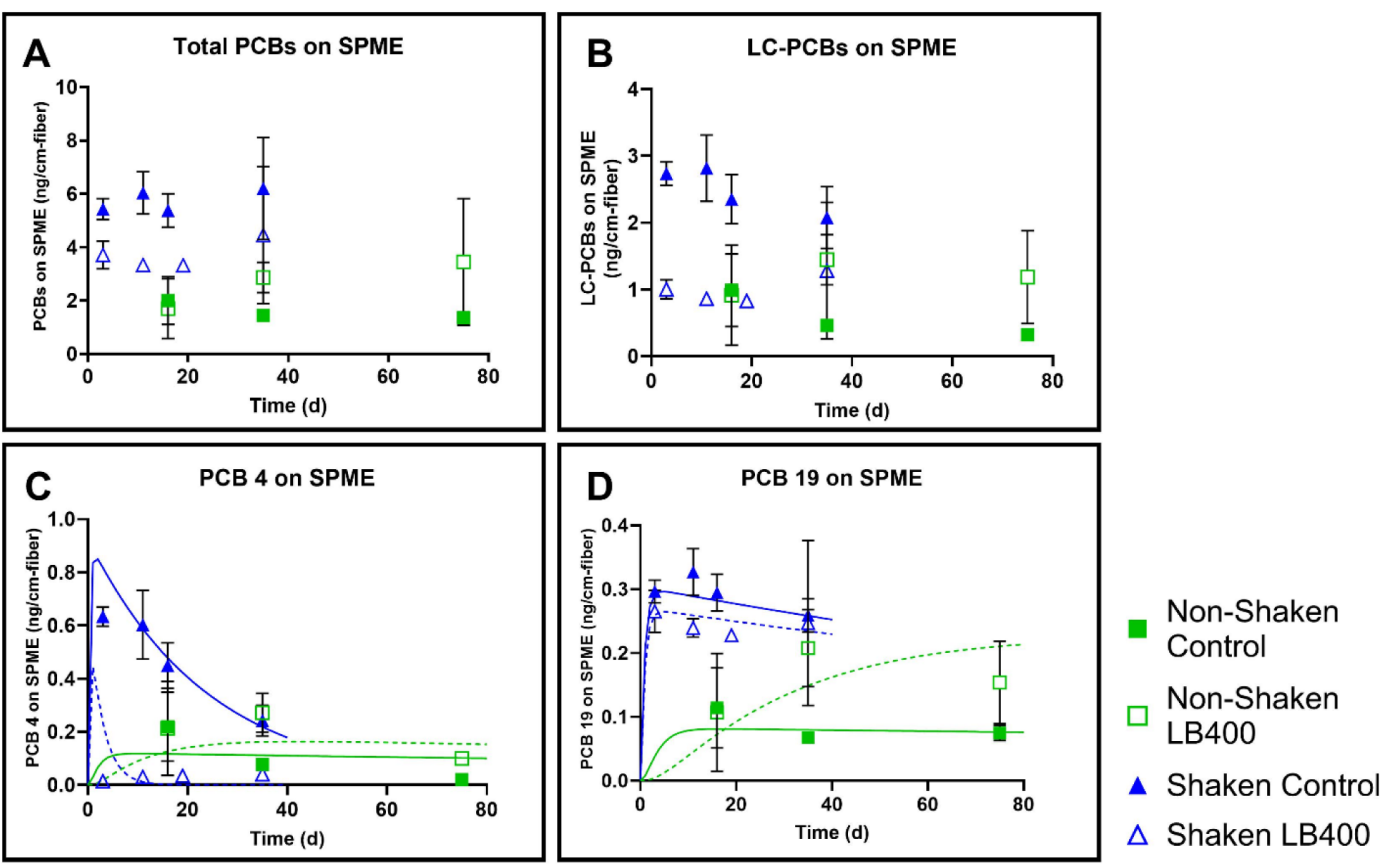
Aqueous phase PCBs were higher in nonshaken LB400-bioaugmented Altavista sediment (AVL_NS) compared to shaken Altavista sediment (AVL_S). Panel A: Total PCBs (all 209 congeners) accumulated on SPME after 35 days (AVL_S; blue triangles) and 75 days (AVL_NS; green squares), Panel B: LC-PCBs. Panel C: PCB 4, Panel D: PCB 19. The data points are the average of 3 replicates and the error bars represent the standard deviation. PCB reactive transport model predictions for PCB 4 and PCB 19 concentrations are shown as solid lines (control; green for nonshaken, blue for shaken) or dashed lines (LB400 bioaugmentation; green for nonshaken, blue for shaken).

After 75 days, total aqueous PCBs (Figure 2A) in LB400-treated AVL_NS samples increased by 143% (*p* = 0.164), and aqueous LC-PCBs (Figure 2B) increased by 265% (*p* = 0.056). This trend was also seen for PCB 4 (Figure 2C) in AVL_NS (*p* = 3 × 10^–5^). PCB 19 (Figure 2D) was not significantly different between treatments and controls in AVL_NS (*p* = 0.082).

### Bioaugmentation with Strain LB400 Decreased Airborne PCBs in Non-Shaken Saline Sediments with Different LC-PCB Content

We hypothesized that bioaugmenting saline (NBH) sediment with LB400 under nonshaken conditions would significantly decrease airborne PCBs, compared to nonbioaugmented controls. NBH sediment is contaminated by a different PCB mixture than AVL sediment (Figure S3). At day 35, airborne PCBs were 44% lower in the LB400 treatments, compared to controls (*p* = 0.003). However, by day 75, total airborne PCBs were 20% lower in the treatments compared to controls (*p* = 0.004) (Figure 3A). Airborne LC-PCB trends (Figure 3B) were similar to total PCBs, with a 50% decrease in airborne PCBs at 35 days (*p* = 0.001), and a 26% decrease at 75 days (*p* = 0.002) in treatments compared to controls.

**Figure 3:**
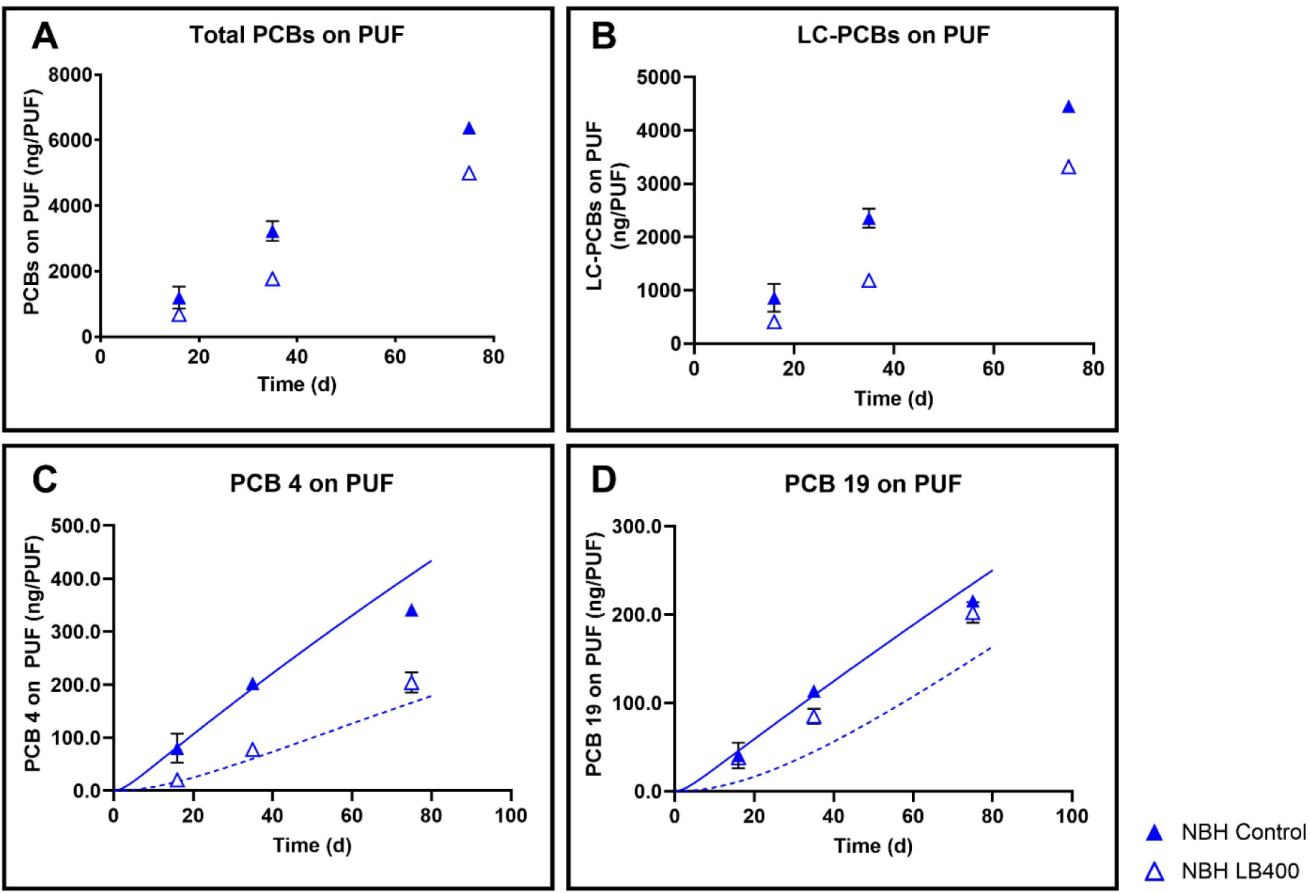
Bioaugmentation with *Paraburkholderia xenovorans* LB400 significantly decreased airborne PCBs from nonshaken New Bedford Harbor (NBH_NS) sediment microcosms. Panel A: Total PCBs (all 209 congeners) accumulated on PUF after 75 days; Panel B: Lower-chlorinated (LC)-PCBs (congeners with <4 Cl atoms; ng/g sediment); Panel C: PCB 4, Panel D: PCB 19. Data points are the average of 3 replicates and the error bars represent the standard deviation. PCB reactive transport model predictions for PCB 4 and PCB 19 concentrations are shown as solid lines (control) or dashed lines (LB400 bioaugmentation).

After 75 days, airborne PCB 4 (Figure 3C) was 40% lower in LB400-treated samples (*p* = 0.003), compared to the control. Similar to total and LC-PCB trends, PCB 4 in air showed greater decreases at 35 days compared to 75 days, with the LB400 treatment being 61% lower (*p* = 0.004) than the control. The decrease in treatment effectiveness of total PCBs, LC-PCBs, and PCB 4 between days 35 and 75 likely indicates decreasing LB400 PCB biodegradation activity with time in the microcosms. Because an auxiliary carbon source was not added in these experiments and bioaugmented LB400 cannot grow on most PCBs in the sediments, a decrease in treatment effectiveness was expected. PCB 19 (Figure 3D) was only 5% lower in the LB400-treated samples in comparison with the controls, which is consistent with our previous observations of LB400’s difficulty in degrading diortho substituted PCBs in AVL sediment.

Interestingly, the difference between the LB400 treatment and the controls was highly significant (*p* = 0.002) in NBH_NS at day 75 and not in AVL_NS (*p* = 0.191), suggesting that LB400 decreased LC-PCB volatilization for longer in NBH sediment than in AVL sediment. AVL (51% LC-PCBs) contained a greater proportion of mono- and dichlorinated biphenyls (40% in AVL and 18% in NBH), particularly PCB 4, while NBH (44% LC-PCBs) had a greater proportion of trichlorinated biphenyls (60% in AVL and 82% in NBH) (Figure S3). Volatilization of the trichlorinated PCBs 18, 20 + 28, 25, 26 + 29, 30, and 31 was lower in the NBH LB400 treatment compared to controls (Figure S4), indicating that LB400 degraded these congeners. Volatilization of PCB 32, a diortho substituted PCB congener like PCB 19, was similar in controls and LB400 treatments over time (Figure S4), indicating it was not degraded by LB400 in NBH_NS sediments, consistent with previous observations in AVL sediments.^23^

Aqueous PCBs in NBH_NS (Figure 4) exhibited the same trend as in AVL_NS (Figure 2), with the LB400-treated samples showing higher aqueous PCBs than the control. After 75 days, total aqueous PCBs (ng/cm SPME) in LB400-treated samples increased by 75% (*p* = 0.017), and aqueous LC-PCBs increased by 91% (*p* = 0.017)), which was not consistent with our hypothesis that aqueous phase PCBs would decrease after adding PCB-degrading strain LB400. This trend was also seen for PCB 4 (*p* = 0.026). PCB 19 on the other hand, was significantly different in NBH_NS (*p* = 0.011) (Figure 4D), unlike AVL_NS (Figure 2D), possibly due to lower variability between replicates.^23^ We believe these trends in both nonshaken experiments were a result of LB400 cell sorption to the SPME fibers due to the lack of mixing in the microcosm. LB400 cells sorbed on fibers can also sorb PCBs, thus overestimating freely dissolved PCB concentrations. We tested this hypothesis with our RTM, yielding predictions that were a better match to the observations.

**Figure 4:**
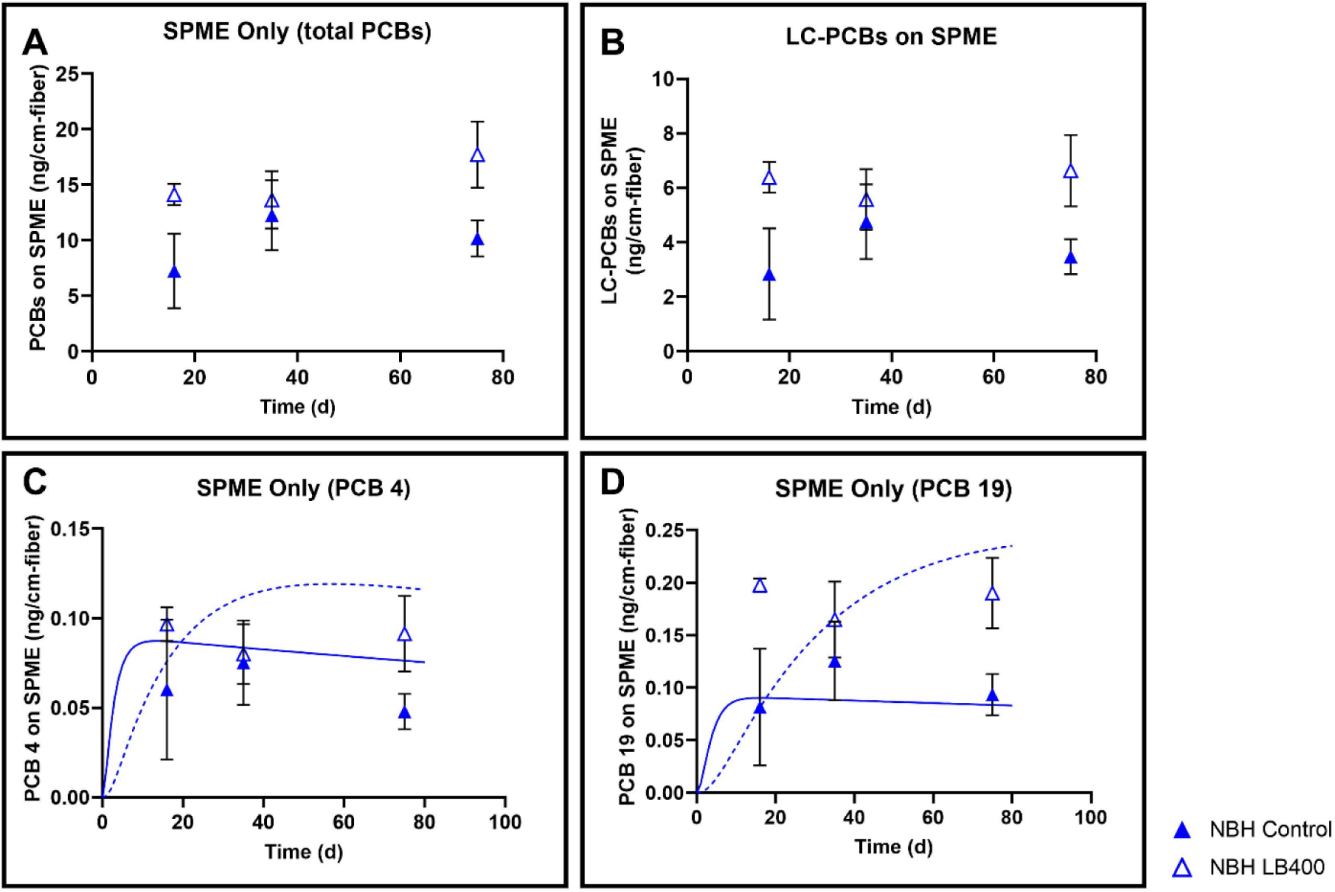
Significantly more aqueous phase PCBs were observed in LB400-bioaugmented nonshaken New Bedford sediment, compared to controls. Mass values are expressed as ng PCB/cm SPME. Panel A: Total PCBs (all 209 congeners) accumulated on SPME after 75 days (AVL_NS; green squares, and NBH_NS; blue triangles), Panel B: Lower-chlorinated (LC) PCBs (congeners with <4 Cl atoms; ng/cm SPME). Panel C: PCB 4, Panel D: PCB 19. The data points are the average of 3 replicates and the error bars represent the standard deviation. PCB reactive transport model predictions for PCB 4 and PCB 19 concentrations are shown as solid lines (control; green for AVL, blue for NBH) or dashed lines (LB400; green for AVL, blue for NBH).

### RTM Performance

The impact of shaking on desorption and sorption processes between sediment and the aqueous or porewater phases and LB400 bioaugmentation were key factors influencing model performance. Overall, the RTM performed better in controls (*R* ^2^ 0.33–0.99). The PUF model (*R* ^2^ > 0.9) outperformed SPME (*R* ^2^ 0.33 to 0.97). Lower *R* ^2^ for SPME could be due to the high variability in the measured values. Sediment type also impacted RTM performance (e.g., PCB 4 SPME *R* ^2^ = 0.83 (NBH_NS) and 0.4 (AVL_NS)). Apart from experimental variability, estimating PCB release from sediment particles to porewater with a radial diffusion kinetic model better described PCB concentrations in NBH sediment. Overall, the model performed well, providing reliable predictions of the various processes occurring and the mass transfer coefficients for these two congeners under different conditions and sediment types.

The presence of LB400 increases system complexity because it biodegrades some PCBs while also acting as a PCB sorbent.^24,73–75^ Our observations suggest that PCB biodegradation and sorption to cells are dynamic, with biological activity and cell concentration changing over time. Since LB400 cell activity was not measured during the experiment, we applied fixed biodegradation rates for PCB 4 (17 d^–1^ for shaken experiments and 0.4–0.6 d^–1^ for nonshaken experiments). This resulted in *R* ^2^ values ranging from <0.1 to 0.88 (SPME) and <0.1 to 0.98 (PUF). Notably, SPME PCB 4 measurements in AVL_S treatments could not be predicted by the RTM (Figure 2C) (i.e., PCB mass accumulated in SPME approximately 70 times lower in treatments compared to the control at 3 days), suggesting that the initial PCB 4 biodegradation rate was much higher than assumed in the RTM. Additionally, PCB 4 biodegradation might have occurred in controls and SPME calibration studies (see details in Section S2), requiring further adjustments to the RTM. For instance, inferred PCB 4 biodegradation rates in controls ranged from 1 to 0.1 (1/d). PCB 4 biodegradation by native microorganisms in the controls is plausible, given the presence of *bphA* in controls (Figure S5).

Accounting for PCB sorption to LB400 cells using the bioavailability factor (B)^47,51^ yielded different results depending on experiment type (shaking vs nonshaking, AVL vs NBH, and PCBs). In shaking experiments, only PCB 19 SPME improved *R* ^2^ (0.88 to 0.97, AVL), while PCB 4 showed no change. PUF showed a minor increase for PCB 4 in AVL (0.1 to 0.2). In nonshaking experiments, SPME showed no improvement for either sediment or PCB, while PUF improved only for PCB 19 in AVL (<0.1 to 0.6). Assuming LB400 cells sorbed to SPME fibers in nonshaking experiments, we modeled this using the bioavailability factor (B) as a surrogate, with a fraction of B sorbed to the SPME fibers. The model improved drastically, with SPME R^2^ for PCB 19 and PCB 4 in both sediments increasing from <0.1 to 0.5. PUF increased for PCB 19 in AVL (<0.1 to 0.6), while other cases remained the same or slightly declined.

### Biphenyl Dioxygenase Gene (*bphA*) Abundance Decreased after 16 Days

We expected *bphA* abundance in LB400-treated samples to decrease by several orders of magnitude after 16 days based on our previous work.^23^ In AVL_S, *bphA* decreased in the treated samples by approximately 1 order of magnitude after day 16 (Figure S5A), while in AVL_NS and NBH_NS, *bphA* abundance decreased by approximately 2.5 orders of magnitude (Figure S5B). While it is not possible to directly compare shaken and nonshaken experiments due to different sampling protocols, our results indicate that the LB400 population declined over time. Decreases in *bphA* abundance after day 16 were not affected by sediment type, although *bphA* abundance increased in NBH_NS at day 75, which did not occur in AVL_NS. The presence of *bphA* in controls, which overall remained steady, indicates that native sediment microbes with *bphA* could potentially contribute to PCB biodegradation. While the effects of LB400 bioaugmentation on the sediment microbiome are still unclear, the apparent increase in *bphA* abundance in LB400-treated samples in NBH_NS could indicate that LB400 bioaugmentation stimulated the growth of native PCB-degrading microorganisms. To investigate whether native PCB-degrading microorganisms are increasing in abundance in response to LB400 bioaugmentation, and which specific microorganisms may be involved, a 16S rRNA and/or a metagenomic sequencing approach could be employed in future research to definitively determine the impact of LB400 bioaugmentation on the sediment microbiome at NBH and elsewhere.

## Conclusions

With our three experiments using freshwater and estuarine sediments, we demonstrated that LB400 bioaugmentation can decrease PCB volatilization from underwater sediments with different LC-PCB content. This has broad implications for future PCB bioremediation research because different PCB mixtures are widely distributed throughout the world.^1,76^ LC-PCBs in particular are more volatile, and present a significant risk to human health via inhalation, therefore, LB400s ability to degrade LC-PCBs is promising for this potential remediation approach.

This is the first study to examine the effects of adding aerobic PCB-degrading bacteria to shaken and nonshaken sediment microcosms on PCB volatilization, which was significantly decreased in both cases. LB400 decreased LC-PCB volatilization from nonshaken sediment. Not shaking sediment microcosms is generally a more realistic approximation of the natural environment. In our shaken experiment, the sediment was completely suspended in the aqueous phase, while in the nonshaken experiment, the sediment and aqueous phases were separate. Not shaking may also decrease oxygen mass transfer rates, however, this did not appear to affect LB400s ability to decrease LC-PCB volatilization in our experiments.

This study is also the first to employ SPME and PUF as passive samplers in a nonshaken environment with bioaugmentation. The RTM fit our data in both the air and aqueous phases and capture the dynamics occurring in both phases. Combining both passive samplers with the RTM is a valuable tool for testing assumptions about the impact of this type of environment and explaining the effects on our results, can be used as an effective framework for future research, offering a robust method for studying similar environmental systems.

Building upon these promising results, more research on treatment delivery methods is needed, particularly for estuarine environments like New Bedford Harbor, where strong currents would render a liquid culture-based remediation approach impractical. Previous laboratory micro- and mesocosm studies have investigated attaching LB400 to an activated carbon material that served as a delivery vehicle for sediment bioaugmentation.^13,26^ An alternative strategy could use LB400 biofilms grown on biochar made from corn kernels,^22^ which would allow similar treatment delivery while potentially helping the LB400 remain active for longer periods of time. Additionally, encapsulating the LB400 biofilms with sol–gel could further increase the long-term viability of LB400 in the field by protecting it from harsh environmental conditions including high salinity, temperature changes, and shear forces.^77^ This is important because in our experiments the difference between the control and treatment grew smaller over time, indicating a potential loss of LB400 activity.

Combining anaerobic organohalide-respiring bacteria (OHRB) with LB400 biofilms on biochar is a potential solution for *in situ* PCB bioremediation, as OHRB reduce higher-chlorinated PCBs under anaerobic conditions into volatile LC-PCBs that can be degraded by LB400 under aerobic conditions. Previous work has investigated bioaugmenting a PCB-dechlorinating OHRB (*Candidatus* Dehalobium chlorocoercia strain DF1) along with aerobic PCB-degrading strain LB400 into contaminated sediments, which resulted in an 80% decrease in total PCB mass in sediment after 120 days,^13^ while a subsequent field-scale trial noted a 52% decrease in the upper 7.5 cm of sediment after 409 days.^16^ Mono- to nonachlorobiphenyls were uniformly degraded in the field study, suggesting that LC-PCBs produced through anaerobic reductive dechlorination of higher-chlorinated PCBs were degraded by LB400.^16^ Volatilization may have contributed to the substantial observed loss in LC-PCBs as highly active OHRB populations might produce LC-PCBs more quickly than LB400s ability to degrade them especially under the low dissolved oxygen conditions that would be expected in sediments. Although the effect of bioaugmenting both anaerobic and aerobic PCB-degrading bacteria on LC-PCB volatilization from sediment is unclear, it should be investigated in future work as potential excess LC-PCB volatilization poses a significant public health risk.

## Supplementary Material



## Notes

Sampling locations (Figure S1), experimental design (Figure S2), K1 medium preparation (Section S1), methods for PCB extraction with ASE, sediment PCB congener profiles (Figure S3), individual airborne LC-PCBs accumulated on PUF over 75 days (Figure S4), *bphA* abundance over time (Figure S5), primers (Table S1), qPCR parameters (Table S2), and PCB RTM parameters (Section S2) (PDF)
