# Supplementary material for: Paraburkholderia Xenovorans Strain LB400 Significantly Decreased Volatilization of Polychlorinated Biphenyls (PCBs) from Freshwater and Saline Sediments
Source: ACS ES T Water. 2025 Sep 19;5(10):5841–51. doi: 10.1021/acsestwater.5c00423 (PMC12519473; doi:10.1021/acsestwater.5c00423)
Supplement: Supplementary file 1 [file ew5c00423_si_001.pdf]

Supporting Information

Paraburkholderia xenovorans strain LB400 Significantly Decreased Volatilization of Polychlorinated Biphenyls (PCBs) from Freshwater and Saline Sediments

David Ramotowski,[†,‡] *Andres Martinez*[†,‡], *Rachel F. Marek*[†,‡]*, Keri C. Hornbuckle*[†,‡], and *Timothy E. Mattes*[†,‡,*]

[†] Department of Civil and Environmental Engineering, University of Iowa, 4105 Seamans Center, Iowa City, Iowa, 52242, United States

[‡] IIHR—Hydroscience and Engineering, University of Iowa, 100 C. Maxwell Stanley Hydraulics Laboratory, Iowa City, Iowa, 52242, United States

[*] **Corresponding Author**: tim-mattes@uiowa.edu; Phone: +319 335 5065, Department of Civil and Environmental Engineering, 4105 Seamans Center for Engineering, University of Iowa, Iowa City IA, 52242, United States

S1. Site Location, Sediment Information, PCB extraction methods, and K1 media recipe

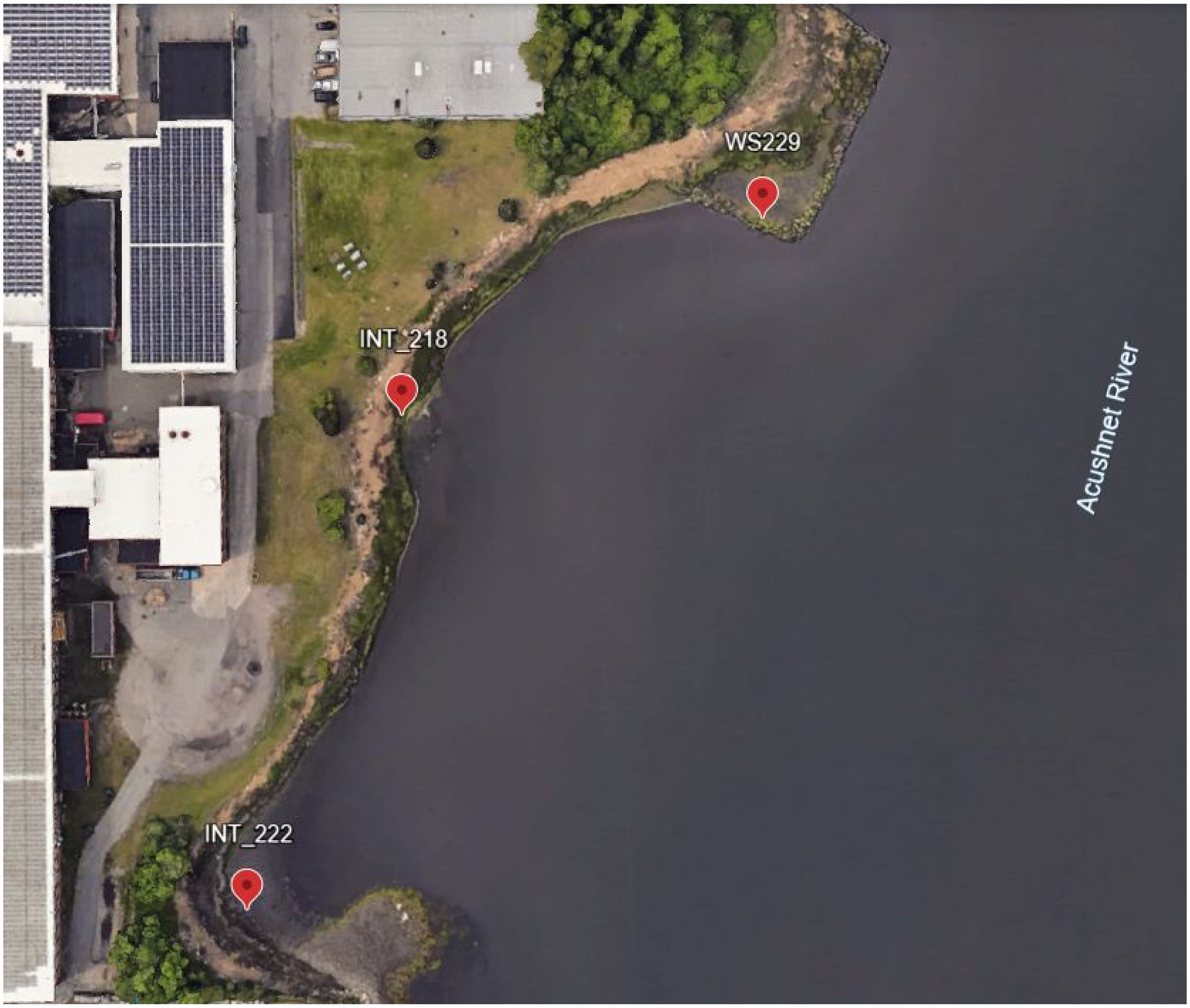

Figure S1. Sample site plan at New Bedford Harbor, Massachusetts, USA.[1] Samples were collected from three locations: INT_222 (41°40'12"N 70°55'09"W), INT_218 (41°40'15"N 70°55'08"W), and WS229 (41°40'17"N 70°55'04"W). Source: Google Earth.

K1 Medium Preparation

A 10x concentrated K1 media stock solution was prepared by adding 15.9 g K_2HPO_4, 2.5 g $(NH_4)_2SO_4$, and 1.77 g NaH_2PO_4 into 1000 mL DI water. This mixture was autoclaved for 30 minutes at 121°C to sterilize. After autoclaving and cooling to room temperature, sterile vitamin solutions were added, consisting of 250 µL thiamine, nicotinic acid, nyo-inositol, and riboflavin (10 mg/mL) in phosphate buffer, and 25 µL of D-biotin in methanol (2 mg/mL).

1x K1 media was prepared by autoclaving 880 mL of DI water for 30 minutes at 121°C, adding 100 mL 10x K1 stock solution, and 20 mL Hutner mix. The 1x K1 media was used for growing LB400 cells and for preparing microcosms.

Hutner mix was made by combing 10 g Nitriloacetic acid (NTA) and ~4-8 KOH pellets to 600 mL sterile DI water and thoroughly mixing until the solution became clear. 14.5 g $MgSO_4 \cdot 7H_2O$, 3.33 g $CaNO_3$, 9.25 mg $(NH_4)_6Mo_7O_{24} \cdot 24H_2O$, 2 g $FeSO_4 \cdot 7H_2O$, and 50 mL Metals 44 solution were then added. After thoroughly mixing with a magnetic stir bar and plate, 400 mL sterile DI water was added to finish preparing 1 L Hutner mix. The metals 44 solution was made by adding 1g EDTA, 4.4 g $ZnSO_4 \cdot 7H_2O$, 2 g $FeSO_4 \cdot 7H_2O$, 0.62 g $MnSO_4 \cdot H_2O$, 119 mg $CaSO_4 \cdot 5H_2O$, 99.4 mg $Co(NO_3)_2 \cdot 6H_2O$, and 70.8 mg $Na_2B_4O_7 \cdot 10H_2O$ to 600 mL of sterile DI water. The ingredients were mixed using a magnetic stir bar and plate, and 400 mL of sterile DI water was added for a final volume of 1 L.

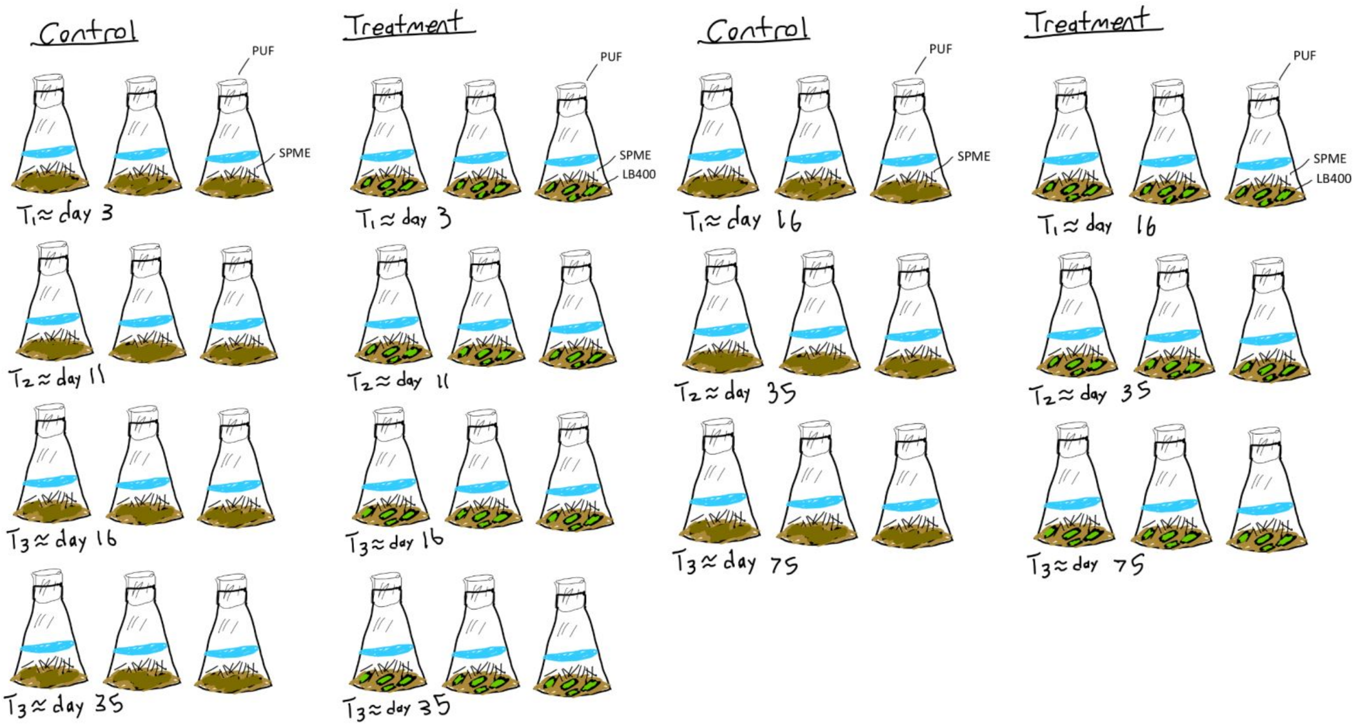

Figure S2. Experimental design matrix for the shaken experiment using Altavista sediment (AVL_S, Panel A), and nonshaken experiments using Altavista and New Bedford Sediment (AVL_NS and NBH_NS, Panel B). Three replicates (n = 3) were used in the control (sediment only) and treatment (*paraburkholderia xenovorans* LB400).

Sediment and PUF PCB Extraction with Accelerated Solvent Extraction (ASE)

PCBs were extracted from New Bedford sediment using an ASE 350 (Dionex, Sunnyvale, California).[2-6] To minimize background PCB contamination, all ASE cells, collection vials, and volumetric glassware were "triple rinsed" with methanol, acetone, and hexane prior to use.

Sediment was combined with diatomaceous earth (DE) using a mortar and pestle until it was a dry powder. The sediment/DE mixture was placed into a 10 mL ASE cell and 9.92 ng of a surrogate standard (248 ng/mL) consisting of one ^{13}C-labeled PCB congener from each of the ten homologs (monochlorinated to decachlorinated biphenyls) was added to account for PCB losses during extraction. A TurboVap II Concentration Workstation (Biotage, Sweden) was used to concentrate sample extracts to ~1 mL. 3.5 mL of hexane was added, and the samples were transferred to test tubes. 2 mL concentrated H_2SO_4 was added, and the samples were inverted for 2 minutes and centrifuged for 5 minutes at 4300 x g. The upper organic solvent phase was transferred to another test tube. 3 mL hexane was added to the first test tube containing the H_2SO_4, and the samples were inverted for 2 minutes and centrifuged for 5 minutes, and the upper phase was transferred to the second test tube. The samples were concentrated to ~1 mL again using the TurboVap II, after which they were eluted with 10 mL hexane through a vertical cleanup column made up of a 22.86 cm (9 in) Pasteur Pipette filled with ~2 cm glass wool, 0.1 g combusted silica gel, and 1 g acidified silica gel (2:1 silica/H_2SO_4 by weight).[7] Samples were again concentrated to ~ 1 mL, transferred into GC vials, spiked with 10 ng of internal standard (deuterated PCB 30, PCB 204; 833 ng/mL), and stored at -10 C prior to running on the GC-MS/MS.

Airborne PCBs were extracted from PUF samples using an ASE 350 (Dionex, Sunnyvale, California) with the following parameters: Heat: 5 min, Static Time: 5 min, Flush Volume (%): 60, Purge Time: 200 sec, Pressure: 1500 psi, Temperature: 100 °C, Cycles: 1, Solvents: 1:1 acetone:hexane. Each PUF was placed into an ASE cell and 9.92 ng of a surrogate standard (248 ng/mL) consisting of one ^{13}C-labeled PCB congener from each of the ten homologs (monochlorinated to decachlorinated biphenyls) was added to account for PCB losses during extraction. A TurboVap II Concentration Workstation (Biotage, Sweden) was used to concentrate sample extracts to ~1 mL. Samples were eluted with 10 mL hexane through a vertical cleanup column made up of a 22.86 cm (9 in) Pasteur Pipette filled with ~2 cm glass wool, 0.1 g combusted silica gel, and 1 g acidified silica gel (2:1 silica/H_2SO_4 by weight).[7] Samples were again concentrated to ~1 mL, transferred into GC vials, spiked with 10 ng of internal standard (deuterated PCB 30, PCB 204; 833 ng/mL), and stored at -10 C prior to running on the GC-MS/MS.

GC-MS/MS operating conditions

The GC operated in solvent vent injection mode under the following injection conditions: initial temperature of 45°C for 0.06 min, ramping at 600°C/min to an inlet temperature of 325°C at 4.4 psi. The GC oven temperature program was as follows: 45°C for 2 min, then increased to 75°C at 100°C/min and held for 5 min, then to 150°C at 15 °C/min and held for 1 min, followed by an increase to 280°C at 2.5 °C/min with a final hold of 5 min (total run time: 70.86 min). The triple quadrupole MS electron ionization source was set to 260°C.

Internal and Surrogate Standard Description

Internal Standard (833 ng/mL):

- Deuterated 2,4,6-Trichlorobiphenyl (PCB D30)
- 2,2’,3,4,4’,5,6,6’-Octachlorobiphenyl (PCB 204)

Surrogate Standard (13C-labelled, 248 ng/mL):

- 4-monochlorobiphenyl (PCB 3)
- 4,4’-dichlorobiphenyl (PCB 15)
- 2,4,4’-trichlorobiphenyl (PCB 28)
- 2,2’,5,5’-tetrachlorobiphenyl (PCB 52)
- 2,3’,4,4’,5-pentachlorobiphenyl (PCB 118)
- 2,2’,4,4’,5,5’-hexachlorobiphenyl (PCB 153)
- 2,2’,3,4,4’,5,5’-heptachlorobiphenyl (PCB 180)
- 2,2’,3,3’,4,4’,5,5’-octachlorobiphenyl (PCB 194)
- 2,2’,3,3’,4,5,5’,6,6’-nonachlorobiphenyl (PCB 208)
- 2,2’,3,3’,4,4’,5,5’,6,6’-decachlorobiphenyl (PCB 209)

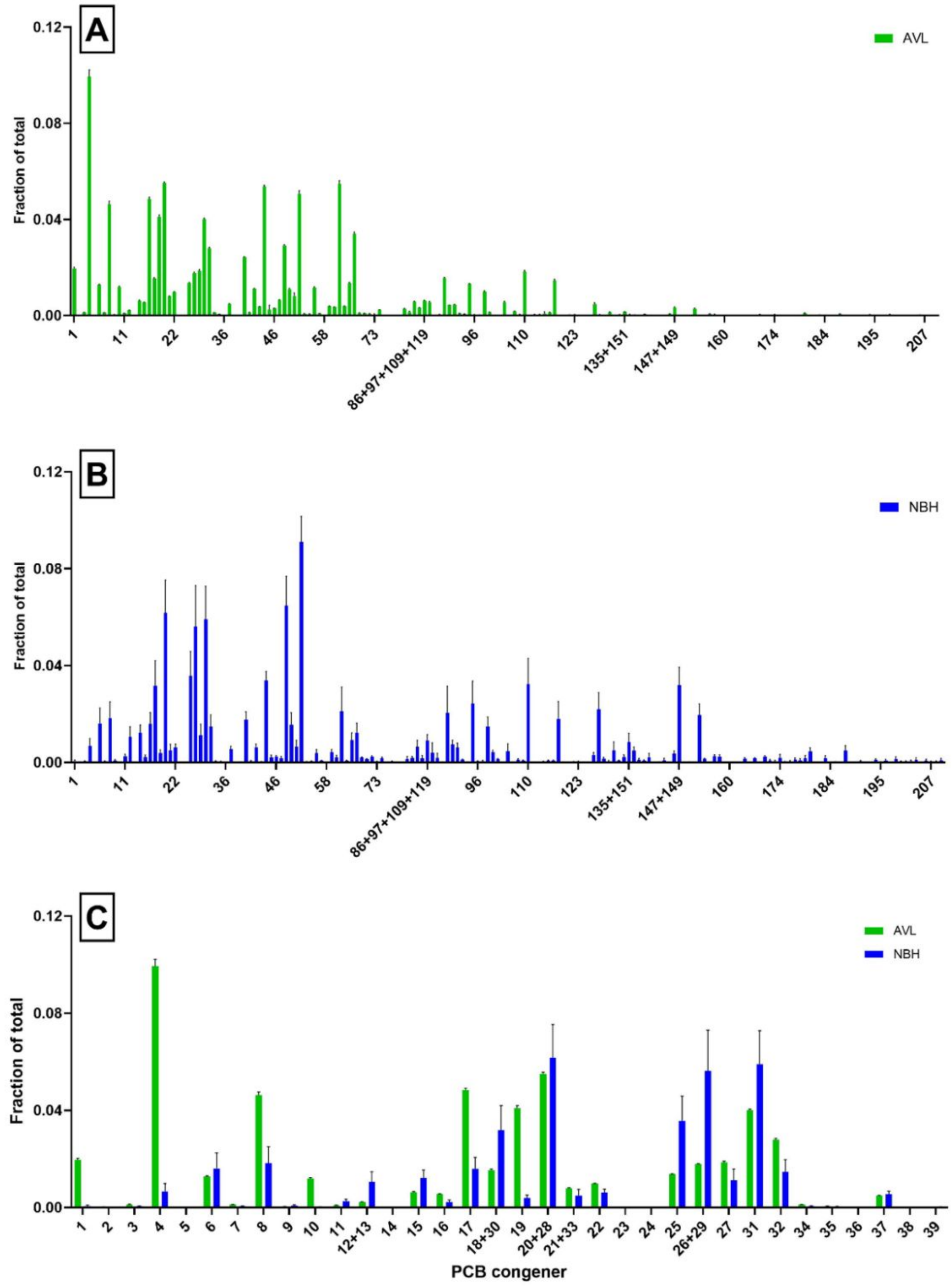

Figure S3. PCB Congener profiles of Altavista (AVL) and New Bedford Harbor (NBH) sediment expressed as a fraction of total PCB concentration (extraction procedure described in S1.3). Panel A: Total PCBs (209 congeners) in AVL. Panel B: Total PCBs in NBH. Panel C: Low-chlorinated (LC)-PCBs, which we defined as PCB congeners with three Cl atoms or less (PCB 1 – PCB 39). Sediment from NBH location INT_222 (Figure S1) was used for our non-shaken NBH microcosm experiment because it had the highest average total (100874 ng PCB g^{-1} dry sediment) and LC-PCB (1351.73 ng g^{-1}) concentrations. Error bars represent the standard deviation of four (AVL) and 11 (NBH) extracted replicates. AVL data was collected by Bako et al. (2022).[8] NBH is contaminated mostly with Aroclors 1242 and 1016 composed of 70% and 60% LC-PCBs, respectively.[4,9-12] Smaller amounts of Aroclors 1254 and 1252 were also used, and corresponding mixtures have been found in NBH.[12,13](2022).[8] NBH is contaminated mostly with Aroclors 1242 and 1016 composed of 70% and 60% LC-PCBs, respectively.[4,9-12] Smaller amounts of Aroclors 1254 and 1252 were also used, and corresponding mixtures have been found in NBH.[12,13]

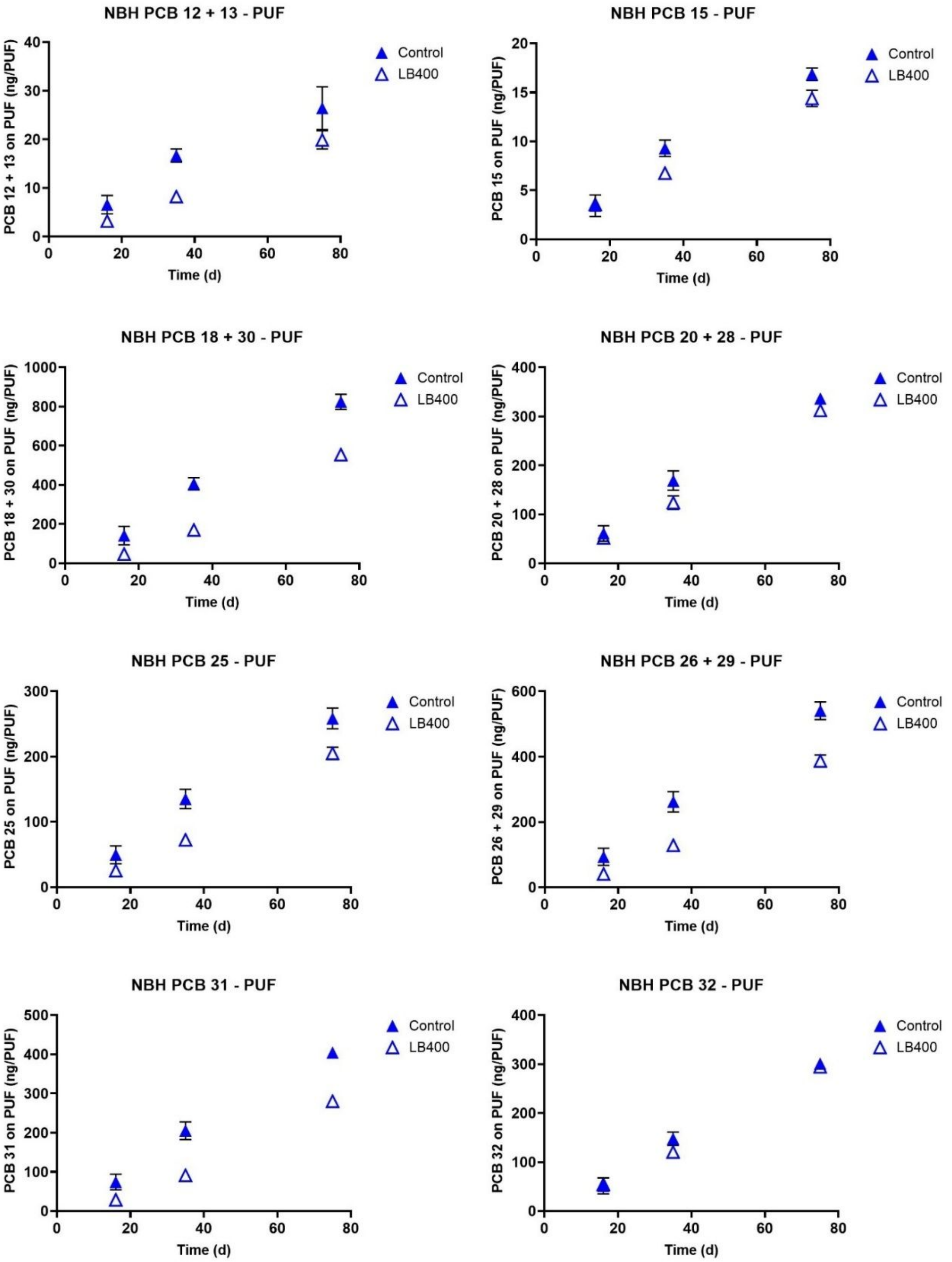

Figure S4. Selected individual airborne LC-PCBs accumulated on PUF over 75 days from NBH_NS. Airborne PCB mass values are expressed as ng PCB per individual PUF. Solid blue triangles: control microcosms with sediment only; open blue triangles: LB400-treated microcosms. Error bars represent the standard deviation of three replicates.

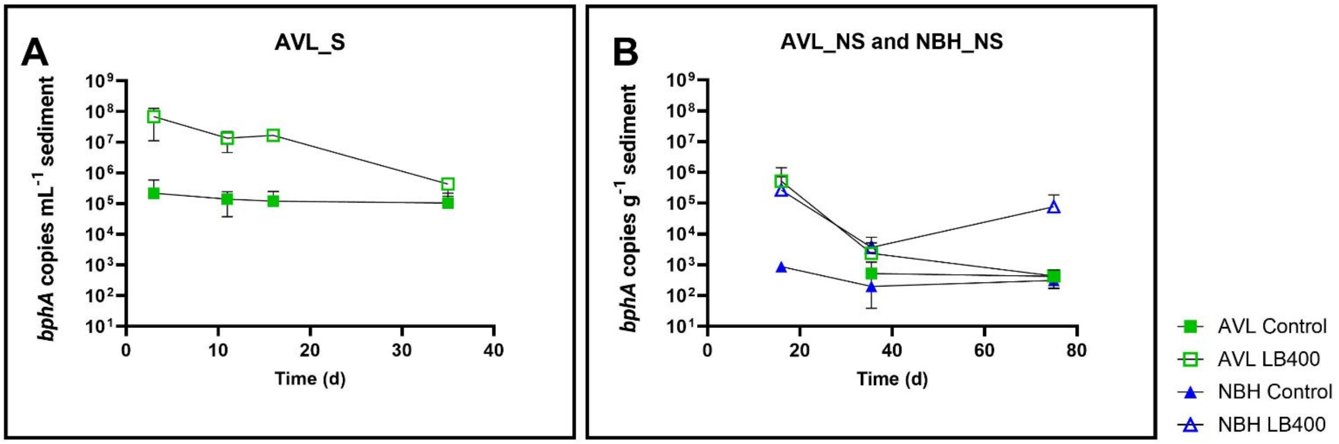

Figure S2. Changes in biphenyl dioxygenase gene (bphA) abundance over time in LB400-treated shaken (AVL_S) and non-shaken (AVL_NS and NBH_NS) microcosms. Panel A: *bphA* copies mL^{-1} sediment slurry in shaken Altavista sediment over 35 days. Panel B: *bphA* copies g^{-1} sediment in non-shaken Altavista (AVL_NS; green squares) and New Bedford Harbor (NBH_NS; blue triangles) sediment over 75 days. The points represent the average of three biological replicates, and the error bars represent standard deviation. The AVL_S samples were shaken at 150 rpm. We were unable to obtain day 16 samples for the AVL_NS control. Data is plotted on a $\log_{10}$ scale.

Table S1. Primer information for the biphenyl dioxygenase subunit A (*bphA*) gene.

**Target gene**	**Primer name**	**Primer sequence (5'-3')**	**qPCR product size (bp)**	**Reference**
bphA	*bphA* 463f	CGCGTSGMVACCTACAARG	211	Petrić et al. (2011)[14]
	bphA 674r	GGTACATGTCRCTGCAGAAYTGC		

Table S2. qPCR Parameters in accordance with MIQE guidelines.

**Experiment**	**Target gene**	**Primer concentration (µM)**	**DNA template Mass (ng)**	**qPCR standard curve range (copies/reaction)**	**qPCR efficiency (%)**	**Y-intercept**
**AVL_S**	*bphA*	1	10	30 – 3 x 10^7	114.775	34.134
**AVL_NS & NBH_NS**					109.697	36.192

S2. PCB Reactive Transport Modeling

PCB 4 and PCB 19 were selected as representative PCB congeners for our modeling purposes. PCB 4 is well known for its degradation in the presence of LB400, while PCB 19 is inert to LB400. Our goal was to capture the primary processes involved in the transport of PCBs through sediment, water, and air systems, including both passive sampler materials. This covered desorption and sorption from sediment particles during mixing, sediment as a bulk phase during non-mixing, and fluxes between the sediment layer, water, and air. Depending on the experimental setup (mixing versus non-mixing), mass balance equations, consisting of ordinary differential equations for each compartment, were developed. We ensured that the mass balance was maintained, meaning the initial mass matched the final mass throughout the modeling period. The physical-chemical properties used to determine partitioning coefficients and mass transfer coefficients for each individual PCB were temperature-corrected. Due to the observed decrease in SPME fiber concentration for certain PCB congeners, including PCB 4 but not PCB 19, during our control AVL_S sediment experiments, we conducted additional SPME uptake experiments using smaller vials with no headspace. Preliminary results indicated that PCB 4 biodegradation is occurring in the AVL sediment, which has been incorporated into our RTM. Additionally, a buffer or bioavailability factor (B) was included for all the treatment experiments. The goal here is to consider LB400 not only as a biodegrading agent for PCBs but also as a sorbent material that reduces the freely dissolved aqueous concentration through sorption.

The following equations apply to the mixing experiments:

$$\frac{dC_s}{dt} = -f \cdot k_{df} \cdot C_s - (1-f) \cdot k_{ds} \cdot C_s + k_a \cdot C_w$$

$$\frac{dC_w}{dt} = f \cdot k_{df} \cdot C_s + (1-f) \cdot k_{ds} \cdot C_s - k_a \cdot C_w - \frac{k_{aw.o} \cdot A_{aw}}{V_w} \cdot \left(C_w - \frac{C_a}{K_{aw.t}}\right) - \frac{k_o \cdot A_f \cdot L}{V_w} \cdot \left(C_w - \frac{C_f}{K_f}\right) - k_b \cdot C_w - k_{LB400} \cdot C_w$$

$$\frac{dC_f}{dt} = \frac{k_o \cdot A_f}{V_f} \cdot (C_w - \frac{C_f}{K_f})$$

$$\frac{dC_a}{dt} = \frac{k_{aw.o} \cdot A_{aw}}{V_a} \cdot \left(C_w - \frac{C_a}{K_{aw.t}}\right) - \frac{r_o \cdot A_{puf}}{V_a} \cdot (C_a - \frac{C_{puf}}{K_{puf}})$$

$$\frac{dC_{puf}}{dt} = \frac{r_o \cdot A_{puf}}{V_{puf}} \cdot (C_a - \frac{C_{puf}}{K_{puf}})$$

Where C_s is:

$$C_s = C_{bs} \cdot M$$

Where C_{bs} is the sediment bulk concentration per mass (ng/g) and M is the solid-water ration (g/L). The bioavailability factor (B) factor was estimated using the initial estimated LB400 cell concentration in the system and the individual PCB equilibrium partition coefficient between the cells and the media (K_{LB400}). The K_{LB400} values were obtained from published PP-LFERs via the UFZ-LSER database website[15], assuming a cellular composition of 60% protein, 5% lipids, 5% phospholipids, and 30% water for LB400. We estimated the initial LB400 cell concentration by assuming that an OD_{600} of 1 is equivalent to 8 x 10^8 cells/mL. Given our final OD_{600} of 0.6 in 100 mL of K1 media, we estimated a starting LB400 concentration of 6.4 x 10^8 cells/mL. We estimated a starting *bphA* gene copy number of 6.4 x 10^{10} copies/100 mL, assuming one *bphA* gene copy per LB400 cell.

The bioavailability factor was calculated as:

$$B = (1 + K_{LB400} \cdot M_{LB400})$$

where K_{LB400} is expressed in liter of water per liter of cell, and M_{LB400} is liter of cell per liter of water. M_{LB400} was derived from the cell concentration per liter and an assumed cell volume of 1 μm^3 per cell. The application of the bioavailability factor (B) was as follows:

$$C_w = \frac{C_w}{B}$$

The following equations apply to the non-mixing experiments:

$$\frac{dC_s}{dt} = -k_s \cdot \left(C_s - C_{pw}\right)$$

$$\frac{dC_{pw}}{dt} = \frac{k_s \cdot V_s}{V_{pw}} \cdot \left(C_s - C_{pw}\right) - \frac{k_{pw} \cdot A_{ws}}{V_{pw}} \cdot \left(C_{pw} - C_w\right) - k_b \cdot C_{pw} - k_{LB400} \cdot C_{pw}$$

$$\frac{dC_w}{dt} = \frac{k_{pw} \cdot A_{ws}}{V_w} \cdot \left(C_{pw} - C_w\right) - \frac{k_{aw.o} \cdot A_{aw}}{V_w} \cdot \left(C_w - \frac{C_a}{K_{aw.t}}\right) - \frac{k_o \cdot A_f \cdot L}{V_w} \cdot \left(C_w - \frac{C_f}{K_f}\right) - k_b \cdot C_w - k_{LB400} \cdot C_w$$

$$\frac{dC_f}{dt} = \frac{k_o \cdot A_f}{V_f} \cdot (C_w - \frac{C_f}{K_f})$$

$$\frac{dC_a}{dt} = \frac{k_{aw.o} \cdot A_{aw}}{V_a} \cdot \left(C_w - \frac{C_a}{K_{aw.t}}\right) - \frac{r_o \cdot A_{puf}}{V_a} \cdot (C_a - \frac{C_{puf}}{K_{puf}})$$

$$\frac{dC_{puf}}{dt} = \frac{r_o \cdot A_{puf}}{V_{puf}} \cdot (C_a - \frac{C_{puf}}{K_{puf}})$$

Where C_s is:

$$C_s = C_{bs} \cdot \frac{\rho_s \cdot (1 - \phi)}{\phi}$$

Where C_{bs} is the sediment bulk concentration per mass (ng/g), ρ_s is sediment density (g/L) and ϕ is the sediment porosity (%). Application of the bioavailability factor (B):

$$C_{pw} = \frac{C_{pw}}{B}$$

$$C_w = \frac{C_w}{B}$$

To model the sorption of LB400 to the SPME fiber, we used the bioavailability factor (B) as a surrogate, with α % of LB400 sorbed to the SPME fiber:

$$C_f = \frac{C_f}{(B \cdot \alpha)}$$

These are the general equations for the equilibrium partition coefficients:

$$K_{xy.t} = K_{xy} \cdot e^{(-\frac{dU_{xy}}{R} \cdot (\frac{1}{T_{w.1}} - \frac{1}{T_{st.1}}))}$$

$$K_{puf} = 10^{(0.6366 \cdot \log K_{oa} - 3.17)}$$

$$K_f = 10^{(10.6 \cdot \log K_{ow.t} - 1.16)}$$

These are the general equations for the mass transfer coefficients:

$$K_{aw.o} = (\frac{1}{K_{aw.a} \cdot K_{aw.t}} + \frac{1}{K_{aw.w}})^{-1}$$

$$k_{pw} = \frac{D_{PCB.w}}{\delta_L}$$

$$k_s = 10^{(-0.832 \cdot \log K_{ow.t} - 1.34)}$$

These are the general equations for the transport properties:

$$D_{PCB.a} = D_{wa} \cdot (\frac{MW_{PCB}}{MW_w})^{-0.5}$$

$$D_{PCB.w} = D_{Co2w} \cdot (\frac{MW_{PCB}}{MW_{Co2}})^{-0.5}$$

$$K_{aw.a} = v_{wa} \cdot (\frac{D_{PCB.a}}{D_{wa}})^{-0.67}$$

$$Sc_{pcb.w} = \frac{v_w}{D_{PCB.w}}$$

$$K_{aw.w} = v_{co2.w} \cdot \left(\frac{Sc_{PCB.w}}{600}\right)^{-0.5}$$

Bioavailability factor (B)

$$B = (1 + K_{LB400} \cdot M_{LB400})$$

Parameter definitions:

C_{bs} = Sediment bulk concentration per mass (ng/g)

C_s = Sediment bulk concentration per volume (ng/L)

C_w = Freely-dissolved water concentration (ng/L)

C_{pw} = Freely-dissolved sediment water concentration (ng/L)

C_a = Air concentration (ng/L)

C_f = SPME fiber concentration (ng/L)

C_{PUF} = PUF concentration (ng/L)

f = fraction of PCBi in sediment particles (unitless)

k_{df} = fast desorption rate from sediment particles (1/d)

k_{ds} = slow desorption rate from sediment particles (1/d)

k_a = sorption rate sediment particles water (1/d)

$k_{aw.o}$ = air-water mass transfer coefficient (cm/d)

A_{aw} = air-water area (cm^2)

V_w = water volume (cm^3)

V_f = SPME volume (LSPME/cm SPME)

$K_{aw.t}$ = Henry's law constant air and water temperature corrected (unitless)

k_o = SPME fiber sampling rate (cm/d)

A_f = SPME fiber area per cm (cm^2/cm)

L = SPME fiber length (cm)

K_f = SPME fiber-water equilibrium partition coefficient (LSPME/Lwater)

k_b = Indigenous biotransformation rate for PCB 4 (1/d)

k_{LB400} = LB400 biotransformation rate for PCB 4 (1/d)

V_a = Headspace volume (cm^3)

r_o = PUF sampling rate (cm/d)

A_{PUF} = PUF base area (cm^2)

K_{PUF}' = PUF equilibrium partition coefficient (m^3/g)

$K_{PUF} = K_{PUF}'$ x d_{PUF} = PUF equilibrium partition coefficient (Lair/Lwater)

V_{PUF} = PUF volume (cm^3)

B = Bioavailability factor (unitless)

K_{LB400} = LB400-water equilibrium partition coefficient (Lwater/LLB400)

C_{LB400} = LB400 volume concentration in water (LLB400/Lwater)

V_{LB400} = LB400 volume (µm3/LB400)

$M_{LB400} = C_{L400}$ x V_{LB400} (LLB400/Lwater)

k_s = sediment-porewater radial diffusion model rate (1/d)

k_{pw} = porewater-water mass transfer coefficient (cm/d)

V_s = Sediment volume (cm^3)

V_{pw} = Porewater volume (cm^3)

A_{ws} = water-sediment area (cm^2)

dU_{xy} = internal energy for the transfer of x-y (J/mol)

Tw.1 = water temperature (K)

Tst.1 = standard temperature (K)

Koa = octanol-air equilibrium partition coefficient

$K_{ow.t}$ = octanol-water equilibrium partition coefficient water temperature corrected

$K_{aw.a}$ = air-side mass transfer coefficient (m/s)

$K_{aw.w}$ = water-side mass transfer coefficient (m/s)

MW = molecular weight (g/mol)

D_{wa} = diffusion coefficient of water in air (cm^2/s)

D_{PCBa} = diffusion coefficient of PCBi in air (cm^2/s)

D_{C02w} = diffusion coefficient of C02 in water (cm^2/s)

$D_{PCB.w}$ = diffusion coefficient of PCBi in water (cm^2/s)

v_{wa}= water velocity of air-side mass transfer coefficient with no air movement (0.003 m/s)

$v_{co2.w}$= C02 mass transfer coefficient in water with no air movement (0.041 m/s)

$Sc_{PCB.w}$ = PCBi Schmidt number

M = solid-water ratio (g/L)

ρ_s= sediment density (1540 g/L)

ϕ= sediment porosity (%)

R = Ideal gas law constant (J/mol/K)

Initial conditions for the mixing experiments were:

$C_s(0) = C_{bs} \cdot M, C_w(0) = C_f(0) = C_a(0) = C_{puf}(0) = 0$

Initial conditions for the non-mixing experiments were:

$C_s(0) = C_{bs} \cdot \rho_s \cdot (1 - \phi)/\phi, C_w(0) = C_f(0) = C_a(0) = C_{puf}(0) = 0$

We calibrated the RTMs using our control experiments, which only differ from the treatment experiments by the presence of LB400. To do this, we calculated the coefficient of determination (R^2) and the residual sum of squares (RSS) between the average observations of the SPME fiber and PUF collected masses at each sampling time and the corresponding model predictions. Our goal was to maximize R^2, ensuring that the model explains as much of the variance in the observed data as possible, while minimizing RSS, indicating minimal discrepancies between the observed and predicted values. These calculations allowed us to estimate the sampling rates for the SPME fiber and PUF, the desorption and sorption rates for the mixing experiments, and the indigenous biotransformation rate for PCB 4, as observed in our control and uptake SPME fiber experiments. Similarly, the same approach was used to estimate the LB400 biotransformation rate in our treatment experiments, but only for PCB 4, as PCB 19 is inert to the presence of LB400.

References

(1) Morris, M. *New Bedford Harbor Superfund Site*; Jacobs: U.S. Army Corps of Engineers New England District, July 2019, 2019.

(2) Bako, C. M.; Martinez, A.; Ewald, J. M.; Hua, J. B. X.; Ramotowski, D. J.; Dong, Q.; Schnoor, J. L.; Mattes, T. E., Aerobic Bioaugmentation to Decrease Polychlorinated Biphenyl (PCB) Emissions from Contaminated Sediments to Air. *Environ Sci Technol* **2022,** *56*, (20), 14338-14349.

(3) Bako, C. M.; Martinez, A.; Marek, R. F.; Hornbuckle, K. C.; Schnoor, J. L.; Mattes, T. E., Lab-scale biodegradation assay using passive samplers to determine microorganisms' ability to reduce polychlorinated biphenyl (PCB) volatilization from contaminated sediment. *MethodsX* **2023,** *10*, 102039.

(4) Bako, C. M.; Mattes, T. E.; Marek, R. F.; Hornbuckle, K. C.; Schnoor, J. L., Biodegradation of PCB congeners by *Paraburkholderia xenovorans* LB400 in presence and absence of sediment during lab bioreactor experiments. *Environ Poll* **2021,** *271*, 116364.

(5) Martinez, A.; Awad, A. M.; Herkert, N. J.; Hornbuckle, K. C., Determination of PCB fluxes from Indiana Harbor and Ship Canal using dual-deployed air and water passive samplers. *Environ Poll* **2019,** *244*, 469-476.

(6) Martinez, A.; Hadnott, B. N.; Awad, A. M.; Herkert, N. J.; Tomsho, K.; Basra, K.; Scammell, M. K.; Heiger-Bernays, W.; Hornbuckle, K. C., Release of Airborne Polychlorinated Biphenyls from New Bedford Harbor Results in Elevated Concentrations in the Surrounding Air. *Environmental Science & Technology Letters* **2017,** *4*, (4), 127-131.

(7) Agency, U. S. E. P., Method 3630C: Silica Gel Cleanup. In US EPA: Washington, D.C, 1996.

(8) Bako, C. M.; Martinez, A.; Ewald, J. M.; Marek, R. F.; Hornbuckle, K. C.; Mattes, T. E.; Schnoor, J. L., Dataset describing polychlorinated biphenyl (PCB) congener accumulation on polyurethane foam (PUF) and solid-phase microextraction (SPME) passive samplers in sediment slurry bioreactors bioaugmented with *Paraburkholderia xenovorans* LB400. **2022**.

(9) Bako, C. M. Mitigating Emissions of Semi-Volatile Polychlorinated Biphenyls (PCBs) from Contaminated Sediments using Aerobic Bioaugmentation. University of Iowa, Iowa City, IA, 2022.

(10) Mattes, T. E.; Ewald, J. M.; Liang, Y.; Martinez, A.; Awad, A.; Richards, P.; Hornbuckle, K. C.; Schnoor, J. L., PCB dechlorination hotspots and reductive dehalogenase genes in sediments from a contaminated wastewater lagoon. *Environ Sci Poll Res* **2018,** *25*, (17), 16376-16388.

(11) Ewald, J. M.; Humes, S. V.; Martinez, A.; Schnoor, J. L.; Mattes, T. E., Growth of *Dehalococcoides* spp. and increased abundance of reductive dehalogenase genes in anaerobic PCB-contaminated sediment microcosms. *Environ Sci Poll Res* **2020,** *27*, (9), 8846-8858.

(12) Weaver, G., PCB contamination in and around New Bedford, Mass. *Environmental science & technology* **1984,** *18*, (1), 22A-27A.

(13) Lake, J. L.; Pruell, R. J.; Osterman, F. A., An examination of dechlorination processes and pathways in New Bedford Harbor sediments. *Marine Environmental Research* **1992,** *33*, (1), 31-47.

(14) Petrić, I.; Hršak, D.; Fingler, S.; Udiković-Kolić, N.; Bru, D.; Martin-Laurent, F., Insight in the PCB-degrading functional community in long-term contaminated soil under bioremediation. *Journal of soils and sediments* **2011,** *11*, 290-300.
(15) Ulrich, N.; Endo, S.; Brown, T. N.; Watanabe, N.; Bronner, G.; Abraham, M. H.; Goss, K. U., UFZ-LSER database v 3.2 [Internet]. **2017**.
